# The Influence of Myelin Oligodendrocyte Glycoprotein on White Matter Abnormalities in Different Onset Age of Drug-Naïve Depression

**DOI:** 10.3389/fpsyt.2018.00186

**Published:** 2018-05-15

**Authors:** Feng Wu, Lingtao Kong, Yue Zhu, Qian Zhou, Xiaowei Jiang, Miao Chang, Yifang Zhou, Yang Cao, Ke Xu, Fei Wang, Yanqing Tang

**Affiliations:** ^1^Department of Psychiatry, The First Affiliated Hospital of China Medical University, Shenyang, China; ^2^Shanghai Mental Health Center, Shanghai, China; ^3^Department of Radiology, The First Affiliated Hospital of China Medical University, Shenyang, China; ^4^Brain Function Research Section, The First Affiliated Hospital of China Medical University, Shenyang, China; ^5^Department of Gerontology, The First Affiliated Hospital of China Medical University, Shenyang, China; ^6^Shenyang Mental Health Center, Shenyang, China

**Keywords:** major depressive disorder, myelin oligodendrocyte glycoprotein, diffusion tensor imaging, inferior fronto-occipital fasciculus, magnetic resonance imaging, onset age

## Abstract

Neurophysiological mechanisms of white matter abnormalities in the earlier onset major depressive disorder (eoMDD, onset age ≤25 years old) differ from that in the later onset MDD (loMDD, onset age >25 years old). Myelin oligodendrocyte glycoprotein (MOG) is an important factor influencing white matter development. The influence of MOG on white matter in MDD of different age onset need to be explored. We compared MOG plasma concentrations and diffusion tensor imaging (DTI) data in 35 first-episode medication-naïve MDD patients (23 eoMDD, 12 loMDD), and 32 healthy controls (HC, 17 younger, 15 older). MOG was significantly higher in eoMDD and lower in loMDD compared with HC. Mean diffusivity (MD) values were significantly increased in inferior fronto-occipital fasciculus (IFOF) in eoMDD, and decreased in loMDD. In both younger and older groups, MOG correlated positively with IFOF MD values. Abnormal MOG has different influence in MDD of different age onset, which is linked to MOG's overly active effect on abnormal white matter in eoMDD and markedly weak effect in loMDD cases. Abnormal MOG would be an important factor in white matter damage in MDD; the influence of MOG differs with onset age.

## Introduction

Previous research (1) has demonstrated that clinical heterogeneity, including the variety of age onsets, is a key factor influencing the treatment of major depressive disorder (MDD). MDD onsets at different ages are assumed to show different clinical symptoms, as well as disease severity and course (2, 3). Convergent evidence suggests that the heritability of MDD onset in the earlier age (eoMDD, onset age ≤25 years old) is influenced by different factors than MDD onset in the later age (loMDD, onset age >25 years old) (4), and that the neurophysiological mechanisms different by age at onset (5–7). Neural development research implied that humans show different neural characteristics at different ages, with brain development rapid, but not totally mature at younger ages, a possible sensitive period (8–14). The brain continues to develop, especially in myelination, until the fourth decade of life, a stable period (9, 15). These principles all imply that age plays an important element of human brain development. Consequently, one might expect neurophysiological differences in the onset of MDD at different ages.

Increasing evidence implicates white matter as an important component of the structural brain changes in MDD (16–18). In the development of the human brain, axon and synaptic pruning would occur quickly, especially in frontal cortex, from adolescence to young adulthood, after which those processes will maintain a stable level (19–23). As one of the myelin associated axon inhibitors (24–29), myelin oligodendrocyte glycoprotein (MOG) is known to limit neurite outgrowth of axon in the maturation of brain, with MOG effects expressed mainly on white matter fibers (30). Although the brain is not mature in adolescence and young adulthood (≤25 years old), because of axon pruning and inadequate myelination, MOG can maintain myelin-axon integrity in normal brain (31). But overexpression of MOG could lead to excessive inhibition of axons (32). In adolescent schizophrenia, errors in pruning localized in frontal cortex has also been demonstrated to be an important element in the pathology of that mental disorder (33). Whether the MOG is also abnormal and leads to axon pruning errors in eoMDD is still unknown.

On the other hand, autopsy studies showed that myelin lesions may exist in adult patients with depression (34), suggesting that myelin lesions may play a key role in the pathology of loMDD (>25 years old). Myelination of human brain gradually increases with age (35) and continues into the fourth decade of life (15, 35–37). The myelin sheaths in the central nervous system (CNS) are produced by oligodendrocytes (38, 39) and also are affected by MOG (36, 40, 41). As a late marker of myelination and oligodendrocyte maturation (31), MOG can concurrently improve the myelination of the CNS (31, 42). This makes information transmission in nerve fibers more rapid and effective, and plays an important role in the maturation of human brain in middle age (15, 37). Lower MOG in more mature adults, accompanied with demyelination, has been found in some nervous system diseases (43–45). Autopsy studies of adult MDD cases also demonstrated decreased MOG gene expression in cortical or subcortical regions of patients' brains (46–48). This suggested that the lower MOG led to a deficit in myelination, which therefore may be a key mechanism in the pathology of loMDD. Based on known differences in the function of MOG at different ages, it seems reasonable to hypothesize that the mechanism of MOG influences MDD differs at different ages of onset. To the best of our knowledge, that age difference has not been explored.

As a factor affecting the development of axons and myelin, MOG may be involved in the white matter abnormalities of MDD; however, the mechanism remains unclear. Diffusion tensor imaging (DTI) is widely used to detect the white matter abnormalities in MDD. Fractional anisotropy (FA) and mean diffusivity (MD) are commonly used to evaluate white matter fiber integrity and microstructure (14, 49–51). We exploited these tools to investigate the effect of MOG on white matter at different ages of MDD onset. Evidence shows that the prefrontal cortex (PFC) is one of the important regions in which MOG is expressed (52). White matter abnormalities of PFC have also been revealed in numerous DTI studies with MDD cases (16, 53–55). Our previous work detected white matter abnormalities in the fornix, which connects the PFC and hippocampus within eoMDD (56). We also found such abnormalities in the superior longitudinal fasciculus (SLF), which connects dorsal lateral prefrontal cortex (DLPFC) and parietal lobe within loMDD (18). These findings suggest that white matter abnormalities in the PFC might be one of the key component of the pathophysiology of MDD. What is not known is what the relationship between white matter abnormalities of PFC and the effect of MOG in eoMDD and loMDD might be; that is an issue we explored in the present research.

In the current study, we recruited the first-episode medication-naïve MDD, and follow up for 6 months to 2 years to exclude the patients turned into bipolar. We measured the concentration of MOG in plasma and FA, MD of white matter of the brain in eoMDD and loMDD, and analyzed the correlation among those measures. We hypothesized that: 1) The concentration of MOG in MDD would differ from those of healthy control participants in different age group; and 2) MOG would be differentially associated with the white matter effects at different ages of MDD onset.

## Materials and methods

### Participants

We recruited 35 patients (23 eoMDD, 12 loMDD) with diagnosed MDD from outpatients at the Department of Psychiatry, First Affiliated Hospital of China Medical University. All adolescent MDD participants were diagnosed by two trained psychiatrists individually using the Schedule for Affective Disorders and Schizophrenia for School-Age Children (KSADS-PL) and met the following inclusion criteria: fulfilling KSADS-PL criteria; first depressive episode; aged 13–17; no comorbid diagnosis of psychosis, bipolar disorder; and no history of psychotropic medication, electroconvulsive therapy or psychotherapy; severity of depression was assessed through the 17-item Hamilton Depression Rating Scale (HAMD-17) (57) and having a score of at least 17 on HAMD-17. All adult MDD participants were also diagnosed by two trained psychiatrists individually using the Structured Clinical Interview for DSM-IV and met the following inclusion criteria: fulfilling DSM-IV criteria for major depressive disorder; having only a single depressive episode; aged 18–45; exhibiting no comorbid Axis I or II diagnosis; no history of psychotropic medication, electroconvulsive therapy or psychotherapy; having a score of at least 17 on HAMD-17. We followed up for 6 months to 2 years, and excluded the patients who subsequently exhibited a bipolar diagnosis.

By means of advertisements in the same region as the hospital, we also recruited 32 healthy control participants, 17 younger healthy controls (yHC) and 15 older healthy controls (oHC) matched for sex, age and education. The Structured Clinical Interview for DSM-IV and the KSADS-PL confirmed the absence of DSM-IV Axis I or II disorders. All the participants were assessed HAMD-17.

Exclusion criteria for all participants included the following: any MRI contraindications; history of head injury or neurological disorder; history of substance abuse or dependence; any concomitant medical disorder. We also excluded participants with a history of mood disorders in their first-degree family members. All participants were right-handed and were scanned within 48 h of initial contact. All participants gave written informed consent, and the adolescent participants' parents or legal guardian provided written informed consent after receiving a detailed description of the study. The study was approved by the Ethics Committee of the China Medical University.

### Procedure

#### Plasma collection

Plasma collection was performed according to standardized protocols. EDTA was used as an anticoagulant. Samples were centrifuged for 10 min at 2,000 × g at 8°C within 30 min of collection and were stored at −80°C until analysis. We used the Bio Trust Specialty Zeal Human MOG ELISA Kit for the determination of MOG concentrations in plasma.

We prepared all standards before starting the assay procedure. All standards and samples were added in duplicate to the Microtiter Plate. We added biotinylated anti-IgG, and combined streptavidin-HRP, TSZ become antibody—antigen—enzyme- antibody complex, after washing completely. We added TMB substrate solution. The intensity of this colored product is directly proportional to the concentration of MOG present in the samples. We measured the optical density (OD) at 450 nm with microtiter plate reader, and calculated MOG concentration by standard curve.

#### MRI data acquisition

Magnetic resonance imaging was performed on a GE Signa HDX 3.0T MRI scanner with a standard head coil at the First Affiliated Hospital of China Medical University, Shenyang, China. DTI was acquired using a spin-echo planar imaging sequence aligned to the anterior commissure-posterior commissure (AC-PC) plane. The diffusion sensitizing gradients were applied along 25 non-collinear directions (*b* = 1000 s/mm^2^), together with an axial acquisition without diffusion weighting (*b* = 0). The scan parameters were as follows: TR = 17,000 ms, TE = 85.4 ms, FOV = 24 cm × 24 cm, imaging matrix = 120 × 120, 65 contiguous axial slices of 2 mm without gap. Participants were instructed to rest with their eyes closed but remain awake during scanning. No participant reported falling asleep during the scan when routinely asked immediately after scanning. The high resolution structural image was acquired using a three-dimensional fast spoiled gradient-echo T1-weighted sequence: TR = 7.1 ms, TE = 3.2 ms, FOV = 24 cm × 24 cm, matrix = 240 × 240, slice thickness = 1.0 mm without gap, 176 slices.

#### MRI data processing and analysis

DTI data were processed by PANDA (Pipeline for Analyzing braiN Diffusion images 1.3.0 http://www.nitrc.org/projects/panda/) software (58), which synthesizes procedures in FSL (http://fsl.fmrib.ox.ac.uk/fsl), diffusion toolkit (http://www.nmr.mgh.harvard.edu/~rpwang/dtk), and MRIcron (http://www.mccauslandcenter.sc.edu/mricro/mricron). Steps are as follows: converting DICOM files into NIfTI images, estimating the brain mask, cropping images, correcting for the eddy-current effect, averaging acquisitions, calculating DTI metrics, and finally, producing diffusion metrics for statistical analysis. The individual images of the diffusion metrics were transformed from native space to a standard Montreal Neurological Institute (MNI) space via spatial normalization (voxel size 2 mm × 2 mm × 2 mm).

#### Statistical analysis

We separated the participants into four groups by age and diagnosis: eoMDD (13–25 years of age), loMDD (26–45 years of age), and their paired HC groups. Two-way analysis of variance (ANOVA) with diagnosis (MDD/HC) and age (eo/lo) as between subject factors was used to compare age, education and HAMD scores with SPSS 22.0 software (SPSS Inc., Chicago, Illinois). Two-sample *t*-test was used to compare illness duration between eoMDD and loMDD groups. MOG concentrations were analyzed using two-way anova tests to detect the age by diagnosis interaction within four groups (*p* < 0.05). Pearson correlations were used to examine the association between MOG and factor score of HAMD.

We also used two-sample *t*-tests to compare the group differences of FA and MD (*p* < 0.005) within the younger groups and older groups in SPM8, extracted the significantly different ROI values, and used the Pearson's correlation to analyze the correlation between MOG concentration and the ROI values. The contrast map threshold was set at *p* < 0.005 for each voxel, with a cluster size of at least 51 voxels, which was equal to the corrected threshold of *p* < 0.05, as determined by AlphaSim (see program AlphaSim by B.D. Ward in AFNI software. http://afni.nimh.nih.gov/pub/dist/doc/manual/AlphaSim.pdf).

## Results

### Demographic and clinical scales

There were no significant effect of diagnosis or sex in age, education and HAMD scores. MDD and HC groups did not differ significantly in age and education. The effect of diagnosis in HAMD was significant, with significant higher HAMD scores in the MDD group, compared to the HC group (*p* = 0.000). There was no significant effect of age in HAMD. Two-sample *t*-test showed no difference in the illness duration between eoMDD and loMDD subgroups (Table 1).

**Table 1:** Demographic and clinical data of subjects.

	**eoMDD**	**loMDD**	**yHC**	**oHC**	**Statistic**	***P*-values**
Number	23	12	17	15	χ^2^ = 1.101	0.294
Age (years, mean ± S.D.) [range]	19.44 ± 4.61 [13–25]	33.77 ± 5.86 [26–44]	18.07 ± 3.85 [13–25]	35.28 ± 6.74 [26–45]	*F* = 2.782	0.100
Education (years, mean±S.D.) [range]	11.5 ± 2.57 [7–16]	11.98 ± 3.52 [7–16]	12.68 ± 3.79 [7–18]	13.21 ± 3.42 [9–19]	*F* = 0.045	0.832
HAMD (mean ± S.D.) [range]	22.09 ± 5.01 [17–33]	21.83 ±5.04 [17–32]	0.88 ± 1.58 [0–6]	0.46 ± 0.96 [0–3]	*F* = 0.013	0.908
Duration of illness (month, mean ± S.D.) [range]	5.75 ± 7.11 [0.5–24]	7.85 ± 7.31 [1–24]	N/A	N/A	*t* = 0.857	0.398
MOG (mean ± S.D.)	214.64 ± 71.47	166.19 ± 79.73	178.71 ± 58.69	208.38 ± 63.11	*F* = 5.198	0.026^*^

*S.D. standard deviation; HAMD, Hamilton Depression Rating Scale; MOG, myelin oligodendrocyte glycoprotein; eoMDD, major depressive disorder onset in the earlier age (onset age ≤25 years old); loMDD, major depressive disorder onset in the later age (onset age >25 years old); yHC, younger healthy controls; oHC, older healthy controls*.

* *P < 0.05*.

### MOG results

The interaction result of age by diagnosis of MOG within four groups was significant (*p* = 0.026). In the younger groups, MOG in eoMDD was significantly higher than in yHC (*p* = 0.010). In the older groups, MOG in loMDD was lower than in oHC (*p* = 0.049; Table 1).

### Correlation between MOG and FA, MD

We excluded participants who failed to scan or yielded bad quality DTI, leaving 34 MDD (22 eoMDD, 12 loMDD) and 30 HC (17 yHC, 13 oHC) participants for analysis.

**Figure 1:**
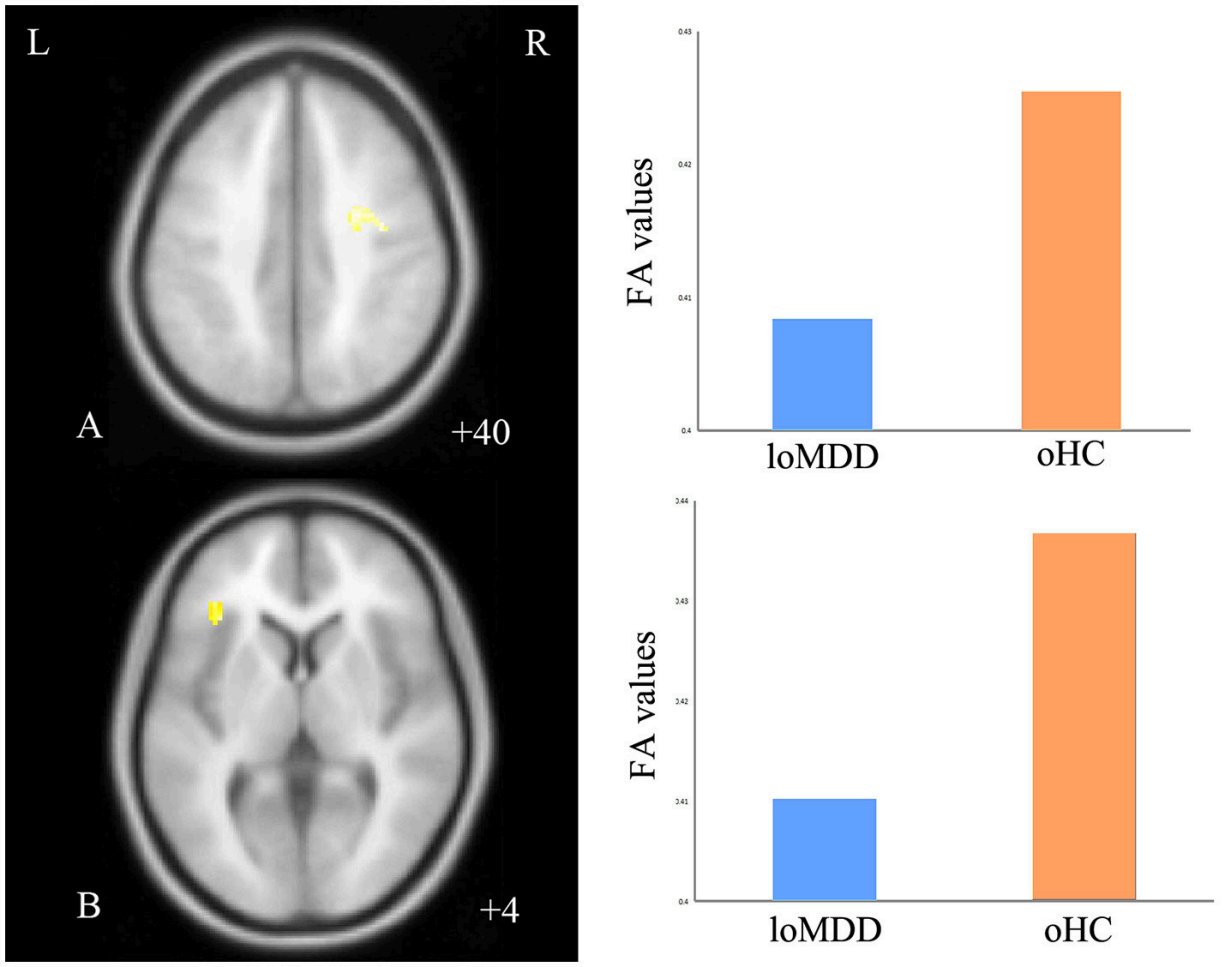
Regions of differences for white matter integrity between medication-naïve patients with major depressive disorder (MDD: eoMDD, onset age ≤25 years old; loMDD, onset age >25 years old) and healthy controls (HC: yHC, younger healthy controls; oHC, older healthy controls). **(A)** The axial image (*z* = +40 mm) shows the significantly decreased fractional anisotropy (FA) values in the right superior longitudinal fasciculus (SLF) with loMDD, compared to oHC. **(B)** The axial image (*z* = +4 mm) shows the significantly decreased FA values in the left inferior fronto-occipital fasciculus (IFOF) with loMDD, compared to oHC. The graph shows FA values for loMDD and oHC.

With respect to FA values, there were no significantly different regions in the eoMDD group relative to the yHC group, but FA was significantly decreased in the right SLF (cluster size = 81 voxels, maximal point MNI coordinate: *x* = 30 mm, *y* = −2 mm, *z* = 40 mm, *T* = 3.07, *p* < 0.05, corrected) (Figure 1A) and left inferior fronto-occipital fasciculus (IFOF) (cluster size = 68 voxels, maximal point MNI coordinate: *x* = −38 mm, *y* = 30 mm, *z* = 4 mm, *T* = 3.35, *p* < 0.05, corrected) (Figure 1B) in the loMDD group relative to the oHC group. The correlation between MOG and FA showed no significant association with FA values in the right SLF and left IFOF of loMDD participants.

**Figure 2:**
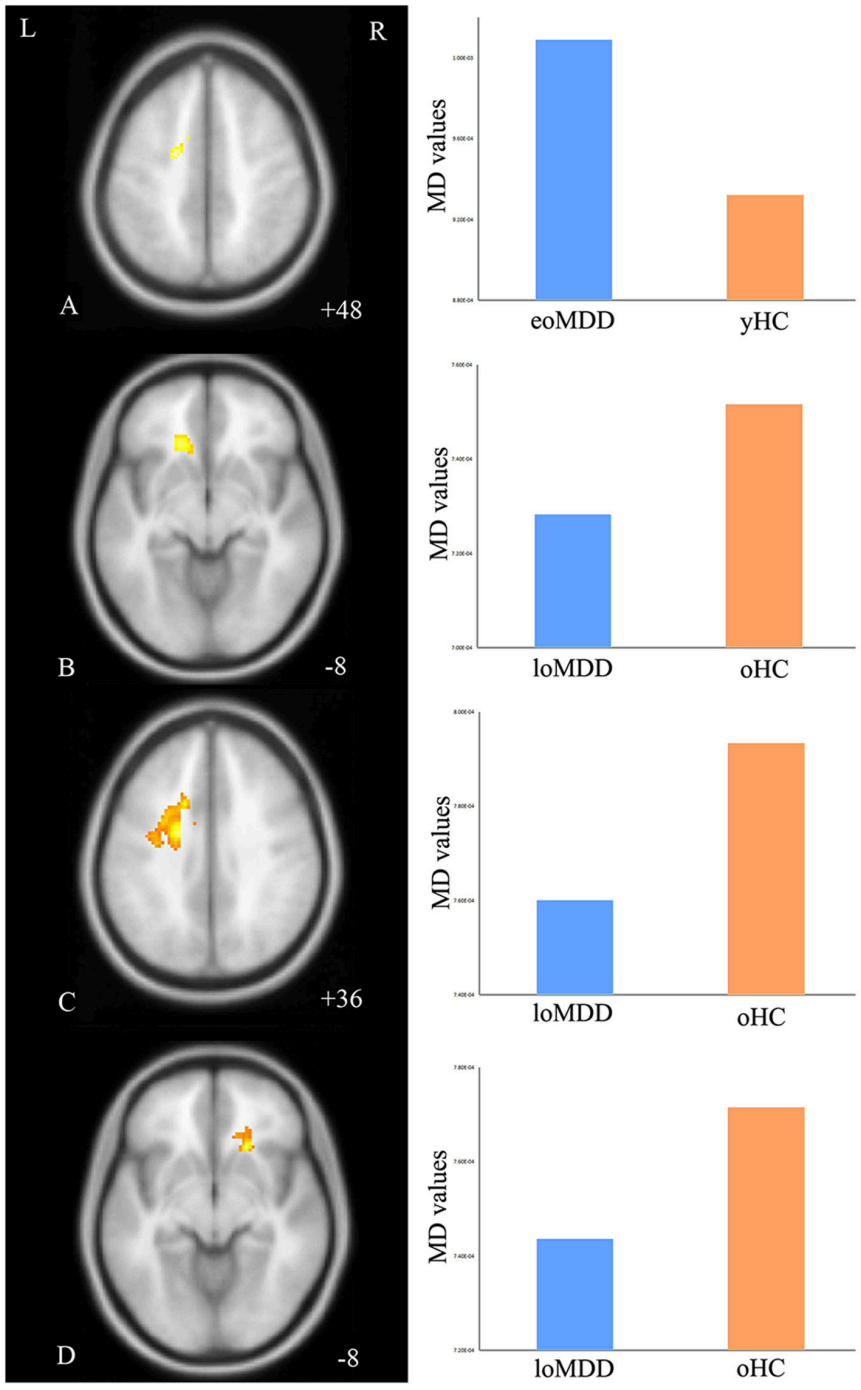
Regions of differences for white matter integrity between medication-naïve patients with major depressive disorder (MDD: eoMDD, onset age ≤25 years old; loMDD, onset age >25 years old) and healthy controls (HC: yHC, younger healthy controls; oHC, older healthy controls). **(A)** The axial image (*z* = +48 mm) shows the significantly increased mean diffusivity (MD) values in the left inferior fronto-occipital fasciculus (IFOF) with eoMDD, compared to yHC. **(B)** The axial image (*z* = −8 mm) shows the significantly decreased MD values in the left IFOF with loMDD, compared to oHC. **(C)** The axial image (*z* = +36 mm) shows the significantly decreased MD values in the left IFOF with loMDD, compared to oHC. **(D)** The axial image (*z* = −8 mm) shows the significantly decreased MD values in the right IFOF with loMDD, compared to oHC. The graph shows MD values for eoMDD, yHC, loMDD, and oHC.

**Figure 3:**
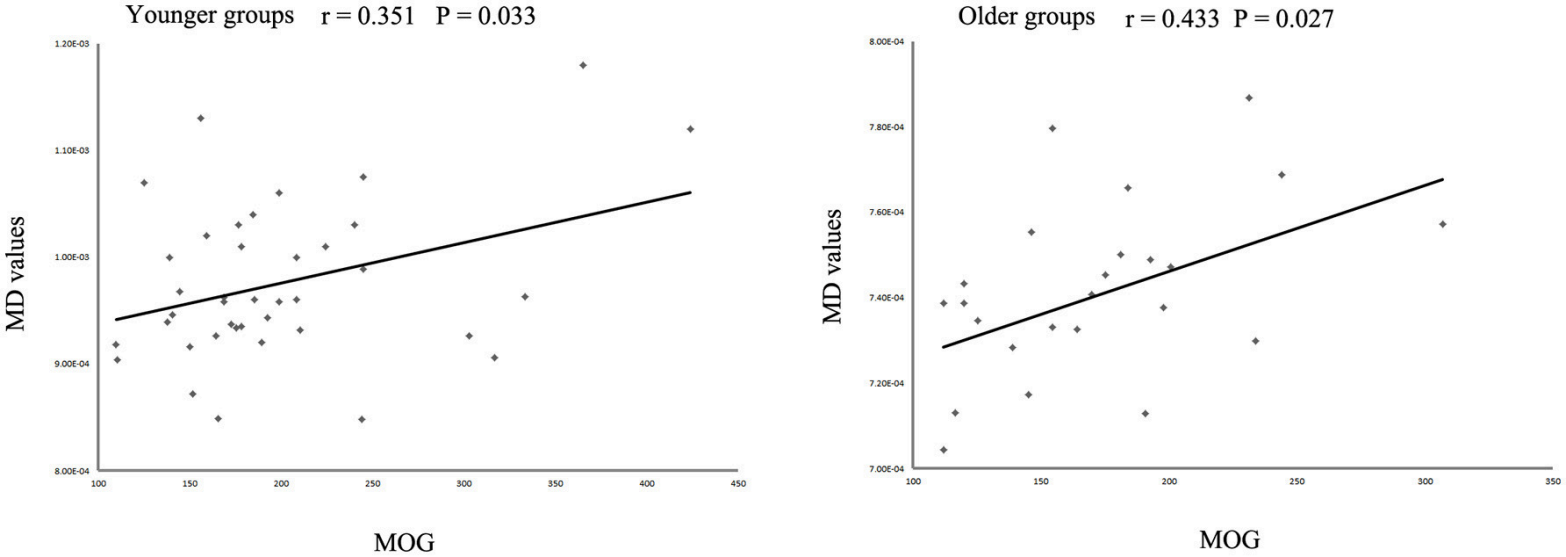
Scatter plots showing significant correlations between myelin oligodendrocyte glycoprotein (MOG) concentration and mean diffusivity (MD) values in the left inferior fronto-occipital fasciculus in different age groups.

The MD values were significantly increased in the left IFOF (cluster size = 79 voxels, maximal point MNI coordinate: *x* = −18 mm, *y* = −2 mm, *z* = 48 mm, *T* = 3.69, *p* < 0.05, corrected) (Figure 2A) of the eoMDD group relative to the yHC group. The ROI values of this region were significantly positively correlated with MOG concentration in younger groups (*r* = 0.351, *p* = 0.033; Figure 3). In older groups, the MD values were significantly decreased in left IFOF (cluster size = 314 voxels, maximal point MNI coordinate: *x* = −18 mm, *y* = 30 mm, *z* = −8 mm, *T* = 4.03, *p* < 0.05, corrected) (Figure 2B), left IFOF (cluster size = 1117 voxels, maximal point MNI coordinate: *x* = −24 mm, *y* = 0 mm, *z* = 36 mm, *T* = 4.58, *p* < 0.05, corrected) (Figure 2C) and right IFOF (cluster size = 605 voxels, maximal point MNI coordinate: *x* = 20 mm, *y* = 28 mm, *z* = −8 mm, *T* = 3.57, *p* < 0.05, corrected) (Figure 2D) in the loMDD group relative to the oHC group. The ROI values of left IFOF (cluster size = 314 voxels, maximal point MNI coordinate: *x* = −18 mm, *y* = 30 mm, *z* = −8 mm) is significantly positive correlated with MOG concentration in older groups (*r* = 0.443, *p* = 0.027; Figure 3, Table 2).

**Table 2:** Brain regions showing significant differences between major depressive disorder and health control participants.

**Brain regions**	**Cluster size (voxels)**	**MNI Coordinates**			***T*-values**
		**X**	**Y**	**z**	
**FA**					
loMDD < oHC					
Right SLF	81	30	−2	40	3.14
Left IFOF	68	−38	30	4	3.35
**MD**					
eoMDD > yHC left IFOF	79	−18	−2	48	3.69
loMDD < oHC					
Left IFOF	314	−18	30	−8	4.03
Left IFOF	1117	−24	0	36	4.58
Right IFOF	605	20	28	−8	3.57

*eoMDD, major depressive disorder onset in the earlier age (onset age ≤25 years old); loMDD, major depressive disorder onset in the later age (onset age >25 years old); yHC, younger healthy controls; oHC, older healthy controls; FA, fractional anisotropy; MD, mean diffusivity; SLF, superior longitudinal fasciculus; IFOF, inferior fronto-occipital fasciculus*.

## Discussion

In the current study, we found MOG to be different by age and MDD diagnosis groups. MOG was significantly higher in eoMDD and lower in loMDD compared with HC. The MD increased in IFOF in eoMDD participants, and decreased in IFOF in loMDD cases. We also found that in older and younger groups, MOG showed a positive correlation with MD values in the left IFOF. To our knowledge, this is the first study to detect the MOG plasma concentration *in vivo* MDD patients, and combining MOG and DTI methods *in vivo* to investigate the relationship between MOG and MD in different MDD onset ages. Our findings imply that the abnormality of MOG may have different mechanism influencing white matter at different ages, thereby inducing different symptoms of eoMDD or loMDD.

MOG is highly expressed on some neurons, particularly on large projection neurons, which is a minor component of central culture medium composed of central nervous system (CNS) myelin (30). Other researchers have recognized that MOG limits neurite outgrowth of axons at younger ages (25–27). By contrast, MOG expression increases with age concurrently with CNS myelination (31), and is a late marker of myelination and oligodendrocyte maturation in adults, independent of axonal influence even in absence of axons (59). There is evidence that myelination of human axons is increased with age (36), with the progression of myelination continuing into the fourth decade of life (15). That perspective is consistent with our result of increased MOG by age in HC, reflecting the increasing myelination in normal brain development.

In our study, we found MOG concentration showed a significant age by diagnosis group interaction, which suggested differential change in MOG at different ages of HC and MDD individuals. We found increased MOG in eoMDD. Overexpression of MOG had been proved leading to excessive inhibition of axons (32). Our result of increased MOG in eoMDD may implicate much axon inhibition or pruning, which may lead to an imbalance in young individuals, and result in depressive symptoms in younger age. Our result of decreased MOG in loMDD is same as the autopsy researches. Autopsy studies showed MOG expression down-regulated in BA 21 (47) of MDD patients. According to the function of MOG in myelination, our result suggested that in adulthood, the lower function of myelination may be an important component of the pathology of loMDD, and possibly leading to widely depressive symptoms. Taken together, our study suggested that the mechanism of MOG influence in the CNS of MDD cases depends on the age of onset. Although CNS of MDD individuals is affected by MOG abnormality, the pathophysiology of eoMDD and loMDD are different. Our study has identified for the first time that MOG in plasma is different between MDD and HC, and showed differential influence for different onset ages. The different pathology of eoMDD and loMDD may relate to the MOG's function: on axon pruning too active at younger ages and on myelination too weak at older ages.

Our study also indicated that the abnormal brain region is more focused in IFOF of MDD cases, compared to HC, in both younger groups and older groups. IFOF connects occipital and frontal lobes, which are involved in reading, attention, and visual processing (60, 61) as well as in semantic processing and conceptualizing of visual stimuli (62, 63). Several studies have reported white matter abnormalities of IFOF in MDD (64, 65). Our results of increased MD value of IFOF in eoMDD, consistent with our previous study (56), suggest that white matter abnormalities of IFOF may play an important role in the pathophysiology of MDD in younger age. Furthermore, decreased FA values of SLF and IFOF were detected in loMDD, which is consistent with our previous study (18). In contrast, our results about decreased MD value of IFOF in loMDD was inconsistent with other studies (65). Until now, the essential association of MD with mental disorders has not been fully understood yet. We speculate that age may play a key role in the different change of IFOF between the two age groups of MDD, which need to be further investigated.

MOG showed a positive correlation with MD values in IFOF within both younger and older groups. This suggested that the abnormal MOG may lead to the abnormality of IFOF, which may play an important role in the pathophysiology of MOG-caused MDD. According to our result, we conclude that the increased MOG in eoMDD may implicate much axon pruning in IFOF, possibly leading to depression in younger age. On the other hand, in loMDD, the decreased MOG's function in myelination may lead to more widely damage of white matter in IFOF, resulted in depression in older age. As the first study exploring the correlation between MOG and DTI *in vivo* in MDD patients, the present results may provide a new direction for the study of the pathology of MDD.

Some limitations of this study should be noted. First, as we selected first-episode medication naïve adolescent and adult MDD to minimize the confounds of chronicity, treatment or comorbidity, the relatively small sample size may limit the generalizability of our results as well as our ability to detect relationships between biomarkers and neuroimaging findings in this study. Future studies with larger sample sizes will be important to further understand the neuropathophysiology of MDD. Second, the MOG and white matter development are both continuous processes from early to late life, the age difference between eoMDD and loMDD group may potentially influence the changes in MOG and its effect on white matter integrity. Future studies between eoMDD and loMDD with matched ages are under investigation and longitudinal study would be needed to further explore the changes in MOG and the effect on white matter in MDD patients. Furthermore, the interpretation of results should be cautious because follow-up studies (66) have found that 20–40% of adolescents with MDD develop bipolar disorder within 5 years after the onset of depression. Although we followed our participants for 6 months to 2 years, longer follow-ups should be done. The cross-sectional design of this study did not allow us to distinguish between trait MDD cases and those who later converted to bipolar disorder; longitudinal studies are needed to examine the difference between them.

In summary, our study of first-episode medication-naïve MDD cases demonstrated that MOG abnormality has different effects at different ages of MDD onset. This influence may be the result of MOG's overly active effect on the abnormal white matter in young individuals and comparatively weaker effect at older ages. These findings suggest that abnormal MOG would be an important factor leading to white matter damage in MDD, with the influence different at different onset ages.

## Ethics statement

The study was approved by the Ethics Committee of the China Medical University. All participants gave written informed consent, and the adolescent participants' parents or legal guardian provided written informed consent after receiving a detailed description of the study.

## Author contributions

FGW, KX, FIW, and YT designed the study. YuZ, QZ, MC, YiZ, and YC acquired the data. FGW, QZ, XJ, and MC analyzed the data. FGW, LK, and FIW wrote the article.

### Conflict of interest statement

The authors declare that the research was conducted in the absence of any commercial or financial relationships that could be construed as a potential conflict of interest.
